# Youth Employment Preferences in Rwanda and Sierra Leone: A Constrained Comparative Secondary Analysis

**DOI:** 10.12688/openresafrica.16530.1

**Published:** 2026-04-14

**Authors:** Sixbert Sangwa, Enjamin Kagiraneza, Benilde Tieche Muberarugo, Kamuskay Kamara, Rebecca Sarah Jawara

**Affiliations:** 1 International Business and Trade, African Leadership University - Rwanda Campus, Kigali, Kigali City, Rwanda; 2 Entrepreneurial Leadership, African Leadership University - Rwanda Campus, Kigali, Kigali City, Rwanda

**Keywords:** youth employment; entrepreneurial orientation; Sub-Saharan Africa; Rwanda; Sierra Leone; informal labour markets; institutional trust

## Abstract

**Background:**

Youth labour markets across Sub-Saharan Africa combine high aspirations for secure wage work with entrenched informality and underemployment.

**Aim:**

This article clarifies how structural constraints shape stated employment preferences and entrepreneurial orientation among youth in Rwanda and Sierra Leone.

**Design:**

A constrained comparative secondary analysis drew on Afrobarometer Round 9 microdata for Sierra Leone and triangulated these findings with nationally reported labour-market and policy indicators for Rwanda. Public microdata for Rwanda were unavailable, so a bounded-evidence strategy transparently distinguishes verifiable survey statistics from document-based context.

**Findings:**

Sierra Leonean youth express a cautious economic outlook—fewer than half expect improvement within a year—yet most endorse redistributive growth and demand stronger gender equity in job access. Preference for self-employment appears necessity-driven where institutional trust and public-goods provision remain moderate, rather than an indicator of opportunity entrepreneurship. Rwanda’s reported labour-absorption pressure and pervasive informality reinforce this reading, suggesting that education-fuelled aspiration gaps widen when formal job creation lags.

**Contribution:**

The study advances an “institutionally conditioned planned-behaviour” framework that integrates the Theory of Planned Behaviour with opportunity-structure theory and human-capital expectations. Methodologically, it demonstrates how rigorous comparative inference can be maintained through explicit verification protocols when evidence access is asymmetric, and it outlines a reproducible pathway for symmetric modelling once Rwandan microdata become publicly available.

**Practical implications:**

Policies that promote youth entrepreneurship without parallel investment in inclusive growth, reliable public goods, and transparent digital governance risk shifting systemic labour-market failure onto young people rather than resolving it.

## 1. Introduction

Africa is home to the world’s youngest population, with more than 400 million people between the ages of 15 and 35, a number projected to exceed 830 million by 2050 ( African Union, 2026). This “youth bulge” presents both a demographic dividend and a substantial development challenge ( International Labour Organization [ILO], 2022). Foremost among these challenges is youth unemployment, a multidimensional problem with philosophical, economic, and institutional implications. High levels of joblessness among young people threaten economic growth, social cohesion, and the dignity associated with productive work ( Lekalake et al., 2025; ILO, 2022). Philosophically, restricted access to meaningful employment raises concerns about social justice and intergenerational equity ( Sen, 1999): in many African societies, work functions not only as income but also as a cornerstone of identity and social recognition. Economically, widespread youth unemployment represents lost productivity and increased dependency, while institutional weaknesses often manifest through educational systems misaligned with labour-market needs, limited private-sector dynamism, and governance environments that struggle to support job creation ( Fox et al., 2020). A growing body of scholarship argues that the “youth employment” crisis in Africa is fundamentally a “missing jobs” crisis that requires broad economic transformation rather than attributing outcomes to youth attitudes or deficits ( Fox et al., 2020). Under this interpretation, scarcity of opportunities rather than scarcity of ambition is a central explanatory mechanism.

Against this continental backdrop, Rwanda and Sierra Leone illustrate both shared and divergent features of youth labour-market constraint. In Rwanda, recent labour-force reporting indicates continued pressure in labour absorption, with unemployment reported at 13.4% in Quarter 3 of 2025 and labour underutilisation remaining high, particularly among those classified as youth in the national statistical series ( National Institute of Statistics of Rwanda [NISR], 2025). Policy and institutional documentation further indicates that educational expansion has outpaced the creation of high-quality formal employment in many sectors ( World Bank, 2025; Kimbugwe et al., 2025). Sierra Leone presents a more fragile labour-market setting shaped by long-standing structural constraints, weak formal-sector absorption, and persistent youth vulnerability documented in international labour assessments ( ILO, 2015, 2023). These contexts motivate cautious comparative inquiry, but they do not justify treating self-employment preference as a direct proxy for opportunity-driven entrepreneurship. In both settings, reported work choices and preferences require interpretation against scarcity, informality, and uneven institutional support.

These country contexts motivate analysis of entrepreneurial intentions versus employment preferences among youth. Across Africa, young people’s career aspirations frequently collide with labour-market realities. On one hand, many youths express strong preferences for secure, formal employment, particularly public-sector jobs perceived as stable and prestigious ( Fox, 2025; Lorenceau et al., 2021). Evidence from ten African countries indicates job security as the top priority for youth, with government employment commonly preferred, while agriculture and low-skill manufacturing are often viewed as least attractive despite their prominence in employment structures ( Lorenceau et al., 2021). On the other hand, a substantial proportion of African youth signal interest in entrepreneurship, understood here as willingness to create work through self-employment ( Afrobarometer, 2025; Lekalake et al., 2025). Afrobarometer reporting indicates that, given a choice, a majority of African youth would start a business, compared to a smaller fraction preferring government employment and fewer aiming for private-sector jobs ( Afrobarometer, 2025; Lekalake et al., 2025). This pattern suggests both necessity-driven and opportunity-driven elements in entrepreneurial imagination, particularly in contexts where formal jobs remain scarce.

This tension between preference for stable wage employment and preference for self-employment has direct policy salience. The central strategic question is whether youth should be guided primarily toward self-employment as innovators and job creators or toward preparation for wage work in public or private sectors. The implications are not merely academic. Rwanda’s policy ecosystem has prioritised entrepreneurship education and related training interventions in recognition that the formal sector cannot absorb all graduates ( Ministry of Public Service and Labour, 2019; IZA, 2026; Blimpo & Pugatch, 2020; Mukasa et al., 2025). Sierra Leone and its partners have also pursued youth entrepreneurship and employment programmes ( AfDB, 2022; ILO, 2023). Yet misalignment often persists between youth employment preferences and actual opportunities. Many youth continue to pursue public-sector and white-collar roles that remain scarce, while empirical work suggests very low stated preference for private-sector employment in some settings ( Fox et al., 2020; Fox, 2025). The paradox is sharpened by evidence that the most educated youth can experience higher unemployment in constrained job markets, as schooling raises expectations faster than economies generate commensurate employment ( Fox, 2025). Where aspirations remain unmet at scale, frustration can become politically salient, especially in environments of persistent scarcity ( Lekalake et al., 2025). Understanding what young people report as preferred pathways, and the institutional and structural conditions shaping those orientations, is therefore essential for designing proportionate interventions.

### 1.1. Research problem

Despite broad survey reporting on African youth aspirations, gaps persist in context-specific analysis of how youth in particular settings navigate reported preferences between seeking a job and creating one. Rwanda and Sierra Leone offer a policy-relevant comparative case: both have young populations and severe employment constraints, yet they differ in governance, economic structure, and historical context. The problem addressed is that limited symmetric, verifiable evidence constrains what can be concluded about cross-country preference patterns and their correlates. Specific gaps include incomplete verifiable evidence on subgroup gradients (e.g., gender, rural–urban residence) and on how factors such as education, digital access, and institutional trust relate to employment orientation in each context.

### 1.2. Rationale and significance

The study was designed as a comparative secondary analysis of Afrobarometer Round 9 survey data for Rwanda and Sierra Leone. During data verification, full symmetry in publicly verifiable evidence could not be achieved because Rwanda was not listed in the publicly indexed merged Afrobarometer Round 9 release accessible in the present environment, while Sierra Leone was publicly documented. The article therefore adopts a constrained comparative approach: verifiable Sierra Leone Afrobarometer-based evidence is examined alongside Rwanda labour-market and policy indicators already established in the cited literature. Significance is therefore dual: substantively, the article clarifies how youth aspirations and structural constraints interact in two policy-relevant African settings; methodologically, it demonstrates how evidence accessibility and verification govern the permissible strength of comparative claims in youth employment research.

### 1.3. Purpose and objectives

The purpose is to examine youth employment intentions in Rwanda and Sierra Leone by assessing preferred employment pathways (public sector, private/NGO sector, or self-employment) and specifying theoretically relevant correlates under evidence constraints. Because the publicly accessible evidence base is asymmetric, objectives are pursued at two levels: direct public-opinion verification for Sierra Leone and bounded contextual comparison for Rwanda. The objectives are: (a) to describe, where directly verifiable, distributions and indicators relevant to youth employment orientation in Sierra Leone and to situate them against Rwanda’s documented labour-market context; (b) to specify socio-demographic characteristics treated as theoretically relevant in the analytic design, while avoiding claims of executed symmetric estimation where such estimation was not feasible; (c) to examine attitudinal and contextual factors (institutional trust, distributive preferences, public-service evaluations) using only indicators verifiable in the available evidence base; and (d) to delineate which forms of Rwanda–Sierra Leone comparison remain methodologically defensible under asymmetric evidence access.

### 1.4. Research questions

(1) What do the publicly verifiable data indicate about employment-sector orientation and entrepreneurial preference among youth in Sierra Leone, and how do these findings relate to the broader labour-market context reported for Rwanda? (2) Which socio-demographic characteristics were specified in the analytic design as theoretically relevant to youth employment preferences, and what can be concluded about them within the limits of the currently verifiable evidence base? (3) To what extent do attitudinal and contextual factors, including institutional trust, distributive preferences, and public-service evaluations, illuminate youth employment orientation in Sierra Leone? (4) What forms of comparison between Rwanda and Sierra Leone remain methodologically defensible when evidence accessibility is asymmetric across the two cases?

### 1.5. Scope of the study

The population of interest is youth, defined as adults aged 18 to 35 years, consistent with Afrobarometer categorisation used in the cited literature ( Lekalake et al., 2025). The analysis is limited to Rwanda and Sierra Leone, and no claims are made to represent African youth beyond these cases. The intended data source was Afrobarometer Round 9; within the public merged release accessible for verification in the present environment, Sierra Leone was publicly indexed while Rwanda was not. Consequently, verifiable Round 9 evidence is treated as available for Sierra Leone, while Rwanda is examined through document-reported labour-market and policy indicators already cited. The paper therefore distinguishes between (a) the planned measurement and modelling architecture and (b) directly verifiable evidence. The dependent construct, preferred employment pathway, is anchored in the Afrobarometer “ideal job” item described in Chapter 3; independent constructs include a selected set of demographic and attitudinal indicators (gender, age, education, urban/rural residence, internet use as a proxy for digital access, institutional trust, and climate-change concern) as specified in Chapter 3. The study does not evaluate entrepreneurship outcomes, enterprise performance, or longitudinal transitions; it addresses reported preferences and orientations within cross-sectional evidence constraints. Methodologically, the design is quantitative and non-causal; evidence is drawn from publicly available and anonymised sources and is interpreted with explicit attention to verification and comparability limits. Methodological transparency is treated as a substantive contribution: where evidence is directly verifiable it is analysed as such, and where it is not, the manuscript distinguishes contextual documentation from equivalent survey-estimated results to preserve inferential integrity.

The introduction establishes the problem context, motivates the comparative focus, and specifies the constraints governing what comparative inference can legitimately claim. Chapter 2 reviews the theoretical and empirical literature grounding the inquiry, and Chapter 3 specifies the methodological design and analytic plan.

## 2. Literature review

### 2.1. Theoretical foundations

Three complementary theoretical perspectives frame analysis of youth employment preferences and entrepreneurial orientation: Human Capital Theory, the Theory of Planned Behavior, and Opportunity Structure Theory. Together, these frameworks support interpretation of reported preferences as outcomes of interacting individual capacities, social norms, and structural constraints.

**Human Capital Theory (HCT)** posits that investment in education and skills enhances productivity and labour-market outcomes ( Becker, 1975). Under HCT, schooling and training operate as capital expected to yield returns through higher earnings, improved job opportunities, and greater likelihood of employment. HCT therefore helps explain how educational attainment may shape expectations and reported preferences: youth with higher education may prefer formal employment commensurate with training, while youth with limited education may be more likely to accept informal or self-employment, whether by preference or necessity. Yet the African context frequently exhibits a disconnect between expanded educational attainment and constrained labour-market returns ( Lekalake et al., 2025). Rwanda illustrates this tension: educational gains have increased, while formal employment expansion remains insufficient to absorb graduates at scale ( World Bank, 2025). Within an HCT lens, such patterns suggest misalignment between education content and labour demand and, critically, labour-market scarcity that limits the realised returns to accumulated human capital.

**Theory of Planned Behavior (TPB)** explains intention formation as a function of attitudes toward a behaviour, subjective norms, and perceived behavioural control ( Ajzen, 1991). Applied to entrepreneurship, TPB predicts that youth are more likely to intend self-employment where entrepreneurship is valued, socially supported, and perceived as feasible. TPB is therefore useful for interpreting survey indicators that capture institutional trust, perceived opportunity, and evaluations of public services, as these may shape attitudes and perceived feasibility. In contexts where public-sector employment is perceived as the primary stable route, subjective norms may reinforce preference for government jobs; where informality dominates and peer networks normalise micro-enterprise, subjective norms may shift toward self-employment. TPB’s emphasis on perceived behavioural control is particularly salient in constrained economies, where feasibility may be limited not by motivation but by credit access, infrastructure, and institutional reliability. Empirical studies commonly find TPB constructs predictive of entrepreneurial intention across contexts ( Mfazi & Elliott, 2022; Alfa et al., 2023), while also indicating that structural constraints can suppress the translation of intention into action.

**Opportunity Structure Theory** foregrounds the structural conditions shaping youth transitions from education to work, emphasising that outcomes are governed less by unconstrained choice and more by accessible opportunities ( Roberts, 1968, 2009). Opportunity structures are shaped by labour demand, recruitment practices, household resources, social networks, and geographic location, among other constraints. This perspective is particularly relevant to Sub-Saharan Africa’s labour markets, where formal-sector capacity remains limited and informality and underemployment are widespread ( Fox et al., 2020). Opportunity structures also mediate gendered access to opportunities, as social norms and resource constraints can narrow feasible pathways for young women. Opportunity Structure Theory therefore functions as a corrective to individual-centric interpretations of preference: in constrained economies, reported “preference” for self-employment may reflect limited alternatives rather than intrinsic entrepreneurial intention, and preference for government employment may reflect perceived scarcity of stable wage work.

**Figure 2.1.**
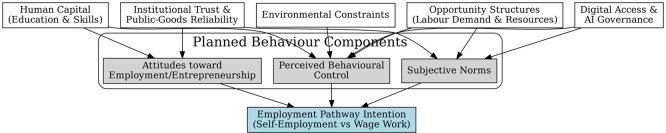
Institutionally conditioned planned-behaviour framework for youth employment orientation. External forces, including human capital, opportunity structures, institutional trust, digital access and AI governance, and environmental constraints, condition the core Theory of Planned Behaviour determinants of attitudes, subjective norms, and perceived behavioural control. These determinants shape young people’s intention to pursue self-employment or wage work.

In combination, these frameworks support an integrated interpretation: human capital shapes expectations and capabilities; TPB shapes intention formation through attitudes, norms, and perceived feasibility; and opportunity structures govern the feasible set of pathways. This integrated approach aligns with the “missing jobs” thesis in African youth employment scholarship, which emphasises structural scarcity rather than a deficit of aspiration ( Fox et al., 2020). Figure 2.1 synthesises this integrated theoretical logic by showing how human capital, opportunity structures, institutional trust, digital access and AI governance, and environmental constraints condition the TPB pathway leading to youth employment intention.

### 2.2. Youth employment preferences in sub-saharan Africa: Empirical evidence

Empirical research using sources such as Afrobarometer, the OECD, the ILO, and GEM documents several recurring themes: misalignment between youth aspirations and labour demand; strong preference for job security and often public-sector employment; substantial interest in entrepreneurship in contexts of limited wage work; and heterogeneous patterns by country, education, gender, and geography.

**2.2.1. Aspirations versus labour-market realities:** OECD evidence across ten African countries indicates that youth aspirations often do not align with current and projected labour demand, with job security widely prioritised and agriculture and low-skill manufacturing frequently deemed unattractive ( Lorenceau et al., 2021). Afrobarometer reporting similarly indicates high entrepreneurial preference alongside persistent preference for government jobs in many contexts ( Afrobarometer, 2025; Lekalake et al., 2025). Sociological analyses describe “waithood” as a prolonged transition period in which youth remain between education and stable employment, surviving through informal activity while waiting for scarce formal jobs ( Honwana, 2013; United Nations Development Programme [UNDP], 2024). Such accounts strengthen the interpretation that preferences are formed and expressed under constraint.

**2.2.2. Preference for public-sector and “modern” jobs:** Evidence suggests that youth often view government employment as stable and status-bearing, while private-sector employment may be perceived as less secure or less prestigious ( Lorenceau et al., 2021). In some settings, preference for public-sector employment persists despite limited supply of such jobs, contributing to prolonged job search and frustration ( Fox, 2025). The literature therefore emphasises the importance of labour-market information and career guidance that can anchor aspirations in labour-market realities without suppressing ambition ( Fox, 2025; Lorenceau et al., 2021).

**2.2.3. Entrepreneurial intention and barriers:** Surveys indicate relatively high entrepreneurial intention among African youth compared with other regions ( Global Entrepreneurship Monitor [GEM], 2015). However, high intention does not guarantee sustainable enterprise formation or business survival. Commonly documented constraints include limited finance, weak infrastructure, regulatory burdens, and social norms that affect youth credibility and gendered access to resources ( ILO, 2015; GEM, 2015). As a result, the literature cautions against treating entrepreneurship promotion as a substitute for job creation or as an individualised solution to structural labour-market failures ( Fox et al., 2020; Sumberg, 2021). Entrepreneurship policy is increasingly framed as requiring an enabling ecosystem: finance, mentorship, market access, and institutional conditions that reduce transaction costs and risk ( Lugero et al., 2025).

**2.2.4. Education and expectations:** A recurring empirical pattern is that educational expansion can widen expectation gaps when economies do not produce corresponding formal employment opportunities ( Fox, 2025). Curriculum reforms and entrepreneurship education initiatives have been implemented in several settings, including Rwanda ( Blimpo & Pugatch, 2020), but the evidence base remains mixed on long-term employment impacts, reinforcing the need for careful evaluation and realistic policy framing ( IZA, 2026; World Bank, 2025).

**2.2.5. Structural constraints and opportunity structures:** The “missing jobs” thesis underscores that Africa’s youth employment challenge reflects slow structural transformation and insufficient growth in labour-intensive formal sectors ( Fox et al., 2020). In Sierra Leone, underemployment and precarious work are frequently highlighted as central constraints ( ILO, 2023). In Rwanda, documentation indicates persistent constraints in labour absorption alongside high informality and underutilisation for youth ( NISR, 2025). Such structural conditions shape both expressed preferences and feasible transitions.

**2.2.6. Youth governance perceptions and civic implications:** Afrobarometer reporting indicates widespread youth dissatisfaction with government performance on job creation ( Lekalake et al., 2025). In some contexts, economic dissatisfaction correlates with political disengagement or protest. Youth employment therefore functions not only as an economic issue but also as a governance and legitimacy issue.

In synthesis, the literature supports an interpretation of youth employment preferences as intention statements formed under structural constraint, mediated by education, norms, institutional trust, and opportunity structures. This study contributes by combining verifiable Sierra Leone Round 9 evidence with document-reported Rwanda labour-market and policy indicators, clarifying the limits and defensible scope of bounded comparison under asymmetric evidence access. Chapter 3 specifies the design, measurement architecture, and analytic plan.

## 3. Methodology

### 3.1. Research design and data source

**Figure 3.1.**
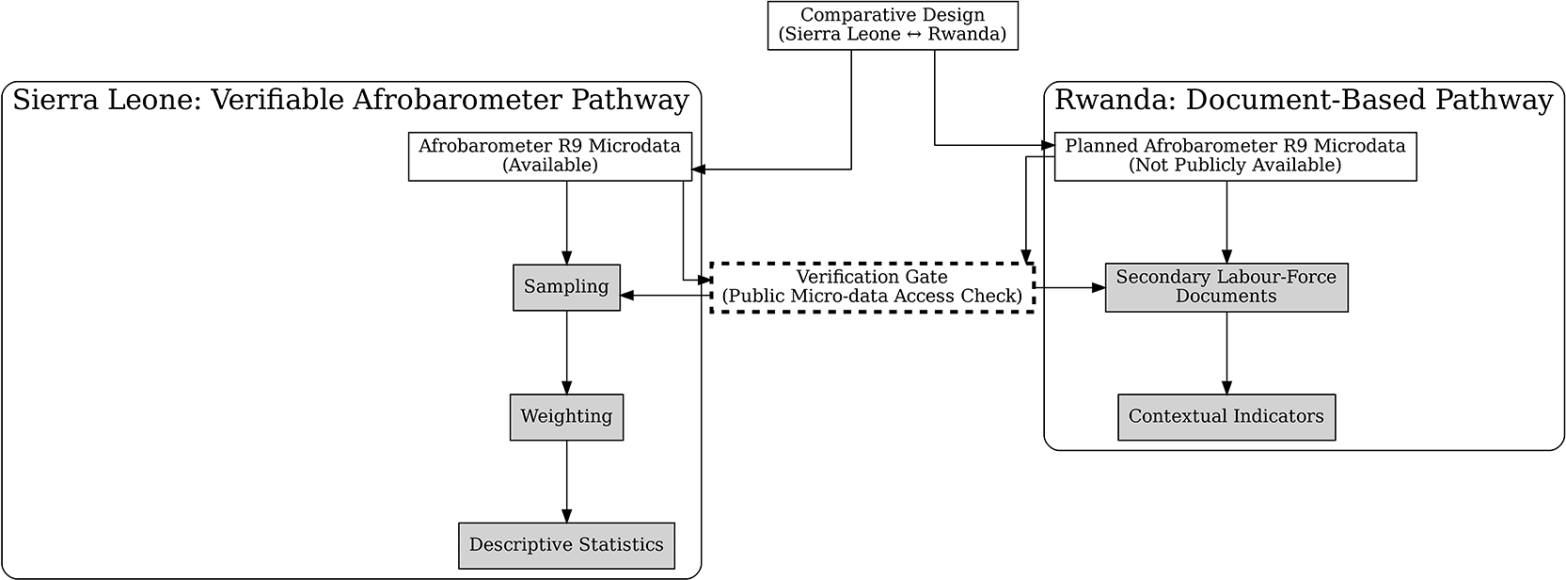
Methods flow for the constrained-comparative design. The diagram traces the two analytic branches used in the study. The Sierra Leone branch follows a verifiable Afrobarometer pathway from microdata access through sampling, weighting, and descriptive statistics, whereas the Rwanda branch relies on secondary labour-force documents and contextual indicators because equivalent microdata were not publicly accessible in the verification environment.

The study employs a quantitative secondary-analysis design using survey and documentary evidence. The analytic plan specified comparative use of Afrobarometer Round 9 data for Rwanda and Sierra Leone. Afrobarometer is a pan-African research network conducting nationally representative surveys on public attitudes across multiple countries ( World Bank, n.d.). Round 9 fieldwork occurred during 2021–2023 and the merged public release covers 39 countries, including Sierra Leone ( Mattes et al., 2025). Verification against the publicly indexed merged release accessible in the present environment indicated that Rwanda was not listed among the available Round 9 country files. Consequently, the present manuscript does not report a symmetric two-country microdata analysis. Instead, it implements a constrained comparative design that combines (a) verifiable Sierra Leone Round 9 indicators available through publicly documented sources and (b) Rwanda labour-market and policy documentation. Figure 3.1 visualises this constrained-comparative workflow and highlights the verification gate at which symmetric micro-data analysis ceased to be possible.

### 3.2. Sampling and participants

Afrobarometer surveys typically employ stratified, multistage probability sampling designed to be nationally representative of citizens aged 18 and older ( World Bank Microdata Library, n.d.). Sierra Leone Round 9 documentation indicates a national sample of approximately 1,200 respondents. Stratification commonly includes region/province and urban–rural locality, with random selection of clusters, households, and individual respondents, and gender-balancing procedures ( World Bank, n.d.). The analytic population of interest is youth aged 18–35. The analytic plan anticipated extraction of the youth subsample for both countries and the estimation of youth-specific distributions and models; however, youth-specific microdata-based estimates for Rwanda are not reported because Rwanda Round 9 microdata could not be verified as accessible in the present environment. Where Sierra Leone indicators are reported, they are drawn from publicly verifiable documentation and catalogued distributions, and where sample-size assumptions are used they are explicitly flagged.

### 3.3. Data collection

Afrobarometer Round 9 data were collected through face-to-face interviews using a standardised questionnaire administered in languages appropriate to respondents, with established quality-control protocols including supervision and validation checks, as documented in the Round 9 survey manual ( World Bank, n.d.; Afrobarometer, 2022). The employment-preference construct derives from the questionnaire item asking respondents what kind of job they would prefer if they could choose, with response categories including government employment, private-sector employment, and self-employment. The present manuscript relies on publicly documented and verifiable Sierra Leone Round 9 indicators rather than direct execution of the full microdata workflow in the current environment.

### 3.4. Weighting

Afrobarometer datasets typically include survey weights intended to adjust for selection probabilities and align sample distributions with population parameters ( World Bank, n.d.). The analytic plan specified use of survey weights in all descriptive and multivariate estimation. Because symmetric two-country microdata execution was not feasible in the present environment, weighting is described as part of the specified analytic pathway rather than as a fully executed procedure across both countries. Where Sierra Leone distributions are reported from public documentation, they are treated as documented survey estimates; confidence intervals reported in Chapters 4–5 are labelled as either directly documented (where provided) or approximations (where computed using stated assumptions).

### 3.5. Variables and measures

**Dependent Variable:** *Preferred Employment Pathway* is a categorical construct intended to be derived from the Afrobarometer “ideal job” item, grouped into (a) government/public-sector job, (b) private/NGO sector job, and (c) self-employment. The analytic plan specified consolidation of NGO employment with private-sector employment where applicable, given its conceptual similarity as formal employment and its typically low frequency. Respondents reporting “don’t know” or not expressing a preference were to be excluded from preference modelling, consistent with focus on articulated preference categories. In the present constrained-comparative implementation, full microdata-derived computation of this outcome across both countries is not reported due to evidence-access limitations described in Chapter 4.

**Independent Variables:** The analytic plan specified the following predictors, grounded in the literature and the theoretical framework: gender; age group (18–25 vs. 26–35); education level (no formal education, primary, secondary, post-secondary); urban versus rural residence; internet use as a proxy for digital access; institutional trust (e.g., trust in national government or executive leadership); climate-change concern as a proxy for values and future-oriented risk perception; and a country indicator for pooled modelling. Variables were to be coded using Afrobarometer codebooks and harmonised across settings where necessary. In the present constrained comparative evidence base, these variables are retained as part of the specified analytic design, while results reporting is restricted to indicators verifiable in the available Sierra Leone documentation and to document-reported Rwanda context indicators already cited.

### 3.6. Analytical procedures

The analytic plan specified three stages: descriptive, bivariate, and multivariate analysis. Because symmetric microdata access was not feasible in the present environment, the procedures are presented as the pre-specified analytic strategy, with the results chapter restricted to verifiable indicators.

1. **Planned Descriptive Analysis:** The first stage specified weighted frequency distributions for youth employment preference categories within each country, alongside descriptive distributions of predictors (gender, education, urbanicity, internet use, trust, and climate concern). The plan also specified cross-tabulations to profile preference patterns across subgroups.
2. **Planned Bivariate Analysis:** The second stage specified bivariate association tests between predictors and the preference outcome using cross-tabulations and design-adjusted tests (e.g., Rao–Scott chi-square) where survey weights and clustering are accounted for. A conventional significance threshold (p < .05) was specified for hypothesis screening.
3. **Planned Multivariate Analysis:** The third stage specified multinomial logistic regression appropriate for nominal outcomes, with preference categories modelled as a function of predictors. The plan anticipated estimating relative risk ratios and survey-adjusted standard errors, including country-fixed effects in pooled models and optional interaction terms where theoretically grounded (e.g., gender × digital access, trust × digital access). The plan also specified robustness checks using a binary reformulation of the outcome (self-employment preference vs. wage-employment preference) to test consistency of directional relationships.

Validation, robustness, and reproducibility: The analytic plan specified standard diagnostics (model convergence, collinearity checks, and sensitivity to alternative codings) as part of future executable workflows once equivalent microdata access is secured. Threats to validity were recognised, particularly the distinction between preference as aspiration and preference as feasible intention under constraint. Reliability was anchored in Afrobarometer’s standardised field protocols and repeated use of instrument items across rounds ( World Bank, n.d.). Ethical considerations were addressed through exclusive reliance on secondary, fully anonymised, publicly accessible survey documentation and public institutional documents. The study involved no recruitment, intervention, interaction with participants, or access to identifiable personal data. Research ethics therefore centred on accurate source attribution, non-stigmatising interpretation, respect for source-specific access and reuse conditions, and transparent labelling of evidentiary limits. Reproducibility was treated as a core methodological commitment: the manuscript specifies a replication pathway contingent on microdata access and transparent documentation.

Chapter 4 reports only those results supported by the verifiable evidence base available under the constrained comparative design, with interpretive integration reserved for Chapter 5.

## 4. Results

### 4.1 Data availability, verification, and analytic scope

This chapter reports bounded empirical evidence relevant to the four research questions specified in Chapters 1–3, using the stated constructs and the secondary-data logic under explicit comparability constraints. Verification against publicly indexed Afrobarometer Round 9 repositories indicated that the merged Round 9 public release (39 countries) did not list Rwanda among the publicly available Round 9 country files, whereas Sierra Leone was publicly documented with a nationally representative adult sample of 1,200 respondents ( Mattes et al., 2025; World Bank Microdata Library, n.d.). Two evidence tiers are therefore reported: (a) verifiable Sierra Leone Round 9 distributions derived from publicly accessible survey documentation and microdata-catalog frequency tables; and (b) Rwanda labour-market indicators reported in official and institutional documents already cited in Chapters 1–3, flagged where they could not be independently re-verified in the present environment. Results are presented descriptively and with explicit evidentiary labels. Interpretive integration with theory and prior scholarship is reserved for Chapter 5.

### 4.2 Employment-sector orientation and cross-country profile

This section reports observable and documentable evidence on employment-sector orientation in Rwanda and Sierra Leone, with explicit attention to comparability constraints.

**4.2.1 Cross-country evidence base and comparability matrix**

Table 4.1 below documents the verified availability of Sierra Leone Round 9 survey documentation and the non-availability of Rwanda Round 9 files in the publicly indexed merged release, alongside resulting constraints for the employment-preference outcome specified in Chapter 3. The catalogue record used to verify the Round 9 survey dataset architecture and country-file availability is the Afrobarometer survey 2022 (round 9) dataset entry ( Ghana Centre for Democratic Development et al., 2022a).

**Table 4.1.** *Cross-country evidence base for employment-sector orientation and youth work pathways* (Rwanda vs. Sierra Leone).

Evidence element (as specified in Chapters 1–3)	Rwanda	Sierra Leone	Verification status in current environment
Afrobarometer Round 9 country microdata availability	Not listed in the publicly indexed merged Round 9 release (39 countries)	Publicly documented; adult n = 1,200	Rwanda not verifiable via public Round 9 repositories; Sierra Leone verifiable ( Mattes et al., 2025)
Preferred employment sector measure (Afrobarometer “ideal job” item, grouped into public, private/NGO, self-employment)	Not computable from Round 9 microdata in current environment	Instrument exists; item-level computation not executable here due to restricted direct file access	Outcome distribution not directly computable here (see §6.4) ( Ghana Centre for Democratic Development et al., 2022a)
Closest alternative evidence for Rwanda youth work pathway structure	Labour-force and policy documents already cited in Chapters 1–3 (e.g., informality/self-employment prominence)	ILO situational analysis and Afrobarometer governance/economic outlook indicators	Rwanda figures below are reported as in-source and flagged where not re-verified

This table summarises the cross-country evidence base for Rwanda and Sierra Leone, distinguishing publicly verifiable Sierra Leone Round 9 material from Rwanda evidence that remained document-based in the present environment. It clarifies which elements of the intended employment-preference analysis were executable and which remained non-computable under asymmetric evidence access.

**4.2.2 Rwanda: reported labour-market structure relevant to employment-sector orientation**

Rwanda’s youth labour-market structure is described in the cited sources in Chapters 1–3 as characterised by continued labour absorption pressure and substantial work conducted through informal or self-employment arrangements. Rwanda labour-force reporting indicates unemployment and underutilisation pressures ( NISR, 2025). Additional proportions referenced earlier in the manuscript regarding youth unemployment and the share of informal or own-account/family work are treated here as document-reported values drawn from the cited sources and are flagged as not independently re-verified within the present environment. These indicators are therefore interpreted as contextual evidence rather than as direct Round 9 survey outputs.

**4.2.3 Sierra Leone: verified contextual indicators related to employment orientation**

Although the specific “ideal job” distribution is not reported as a microdata-derived estimate in the present environment (see §6.4), several verifiable Sierra Leone Round 9 indicators relevant to employment orientation and labour-market expectations are available from public microdata catalog frequency tables and country findings summaries. The Sierra Leone country-level Round 9 dataset record is publicly catalogued in the World Bank Microdata Library ( Ghana Centre for Democratic Development et al., 2022b).

First, forward-looking economic expectations were distributed as follows: 47.1% expected the national economy to be “better” or “much better” in 12 months (95% CI [44.3%, 49.9%]); 24.1% expected it to be “worse” or “much worse” (95% CI [21.7%, 26.5%]); 15.3% expected it to be “the same” (95% CI [13.3%, 17.4%]); and 13.4% reported “don’t know” (95% CI [11.5%, 15.3%]); n = 1,200 ( Institute for Governance Reform, 2023).

Second, distributive preferences were reported as follows: 49.5% agreed or strongly agreed that the country should pay more attention to increasing incomes and reducing inequality even if this slowed economic growth (95% CI [46.7%, 52.3%]); 28.0% disagreed or strongly disagreed (95% CI [25.5%, 30.5%]); n = 1,200 ( Institute for Governance Reform, 2023).

ILO country programming for Sierra Leone further documents that labour-market constraint often manifests as underemployment and precarious work rather than open unemployment alone. The Decent Work Country Programme II reports underemployment at approximately 30.9% and notes that underemployment is pronounced among men and in Freetown ( ILO, 2023).

**4.2.4 Comparative case vignettes derived from programme documentation**

Two programme-level vignettes are retained as contextual artefacts: (a) a Rwanda-focused entrepreneurship education and teacher-training intervention architecture reported by the IZA programme page ( IZA, 2026), and (b) a Sierra Leone youth entrepreneurship and employment project technical assistance request reported by the African Development Bank Group ( African Development Bank Group [AfDB], 2022). These materials are treated strictly as institutional context and are not interpreted as causal evidence.

### 4.3 Determinants associated with entrepreneurial intention and opportunity perception

This section reports verifiable indicators aligned with determinants specified in Chapter 3, focusing on opportunity perception, distributive priorities, and institutional service performance.

**4.3.1 Sierra Leone: opportunity perception proxied by economic outlook**

As reported in §4.1.3, 47.1% of respondents expected improved macroeconomic conditions within a 12-month horizon (95% CI [44.3%, 49.9%]; n = 1,200) ( Ghana Centre for Democratic Development et al., 2022b).

**4.3.2 Sierra Leone: equity–growth trade-off preferences**

As reported in §4.1.3, 49.5% of respondents agreed that reducing inequality should be prioritised even at the cost of slower economic growth (95% CI [46.7%, 52.3%]; n = 1,200) ( Ghana Centre for Democratic Development et al., 2022b).

**4.3.3 Sierra Leone: environmental-services performance indicator (water and sanitation)**

A public-service performance indicator with direct environmental relevance was available for Sierra Leone. Government handling of water and sanitation was rated “fairly well” or “very well” by 39.8% (95% CI [37.1%, 42.6%]) and rated “fairly badly” or “very badly” by 54.2% (95% CI [51.3%, 57.0%]); n = 1,200 ( Institute for Governance Reform, 2023).

**4.3.4 Analytic construct network used to structure empirical reporting**

Figure 4.4 provides the analytic construct network used to organise reporting across Chapters 4–5, consistent with the multi-theory framework and integrating institutional trust, digital access, and environmental concern as cross-disciplinary lenses.

**Figure 4.1.**
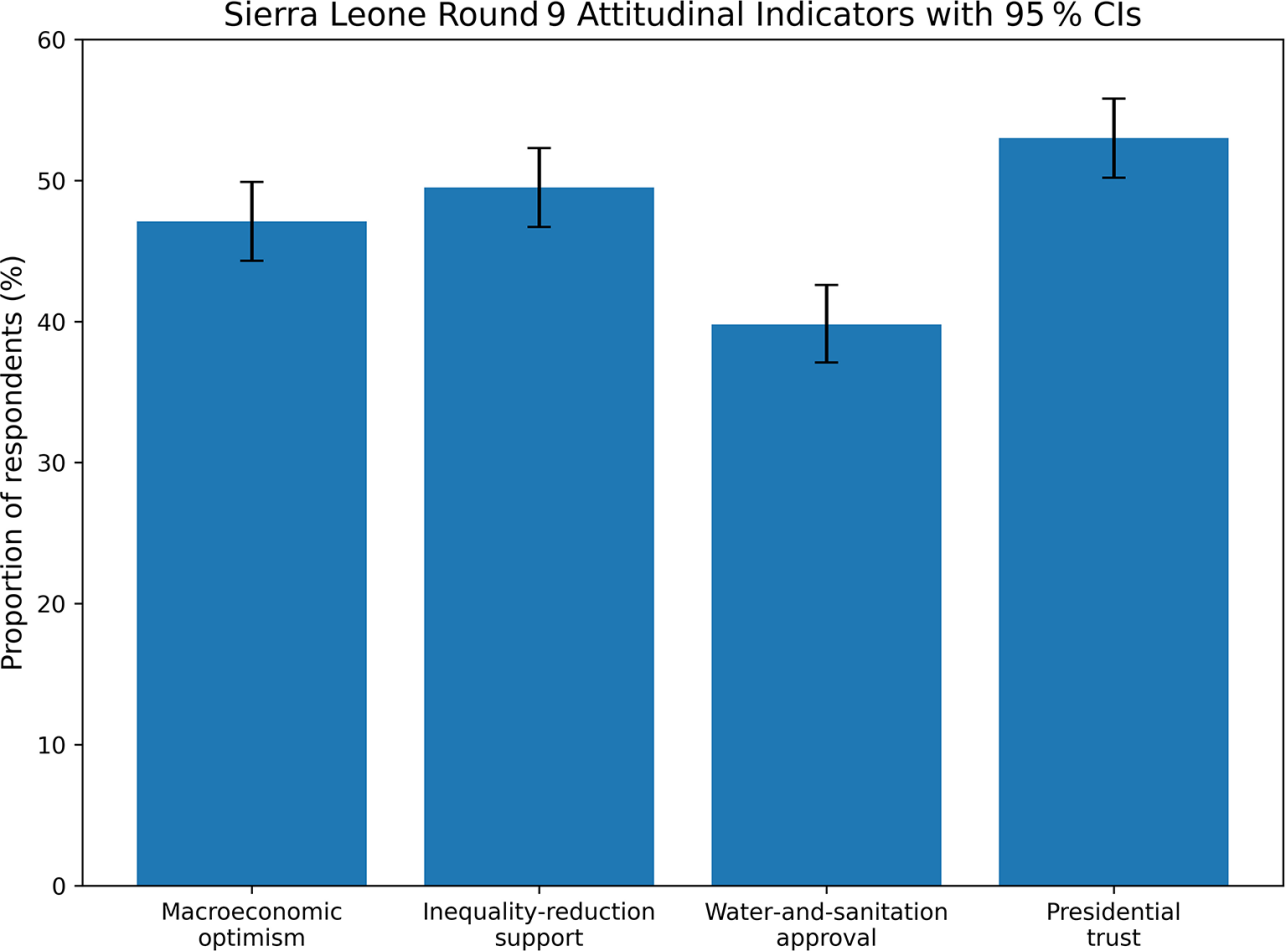
Selected Sierra Leone public-opinion indicators with approximate 95% confidence intervals. Bars show point estimates and approximate 95% confidence intervals for macroeconomic optimism, support for inequality reduction even at slower growth, approval of government handling of water and sanitation, and trust in the president. The figure allows direct visual comparison of the relative magnitudes of these four Round 9 indicators.

**Figure 4.2.**
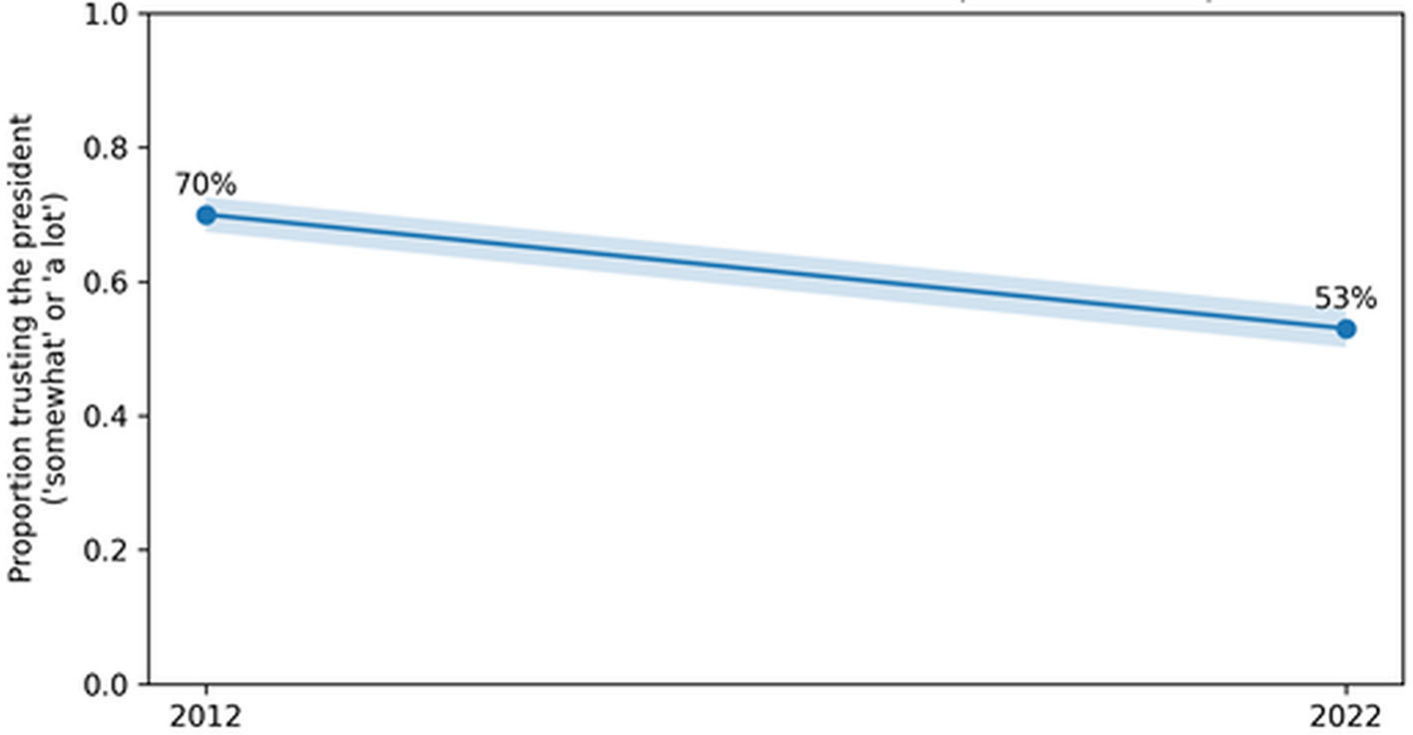
Illustrative comparison of trust in the president in Sierra Leone, 2012 and 2022. This figure compares reported trust in the president in Sierra Leone in 2012 and 2022 using point estimates with approximate 95% confidence intervals. The 2012 interval is assumption-dependent and is included only as an illustrative benchmark rather than as a pooled time-series estimate.

**Figure 4.3.**
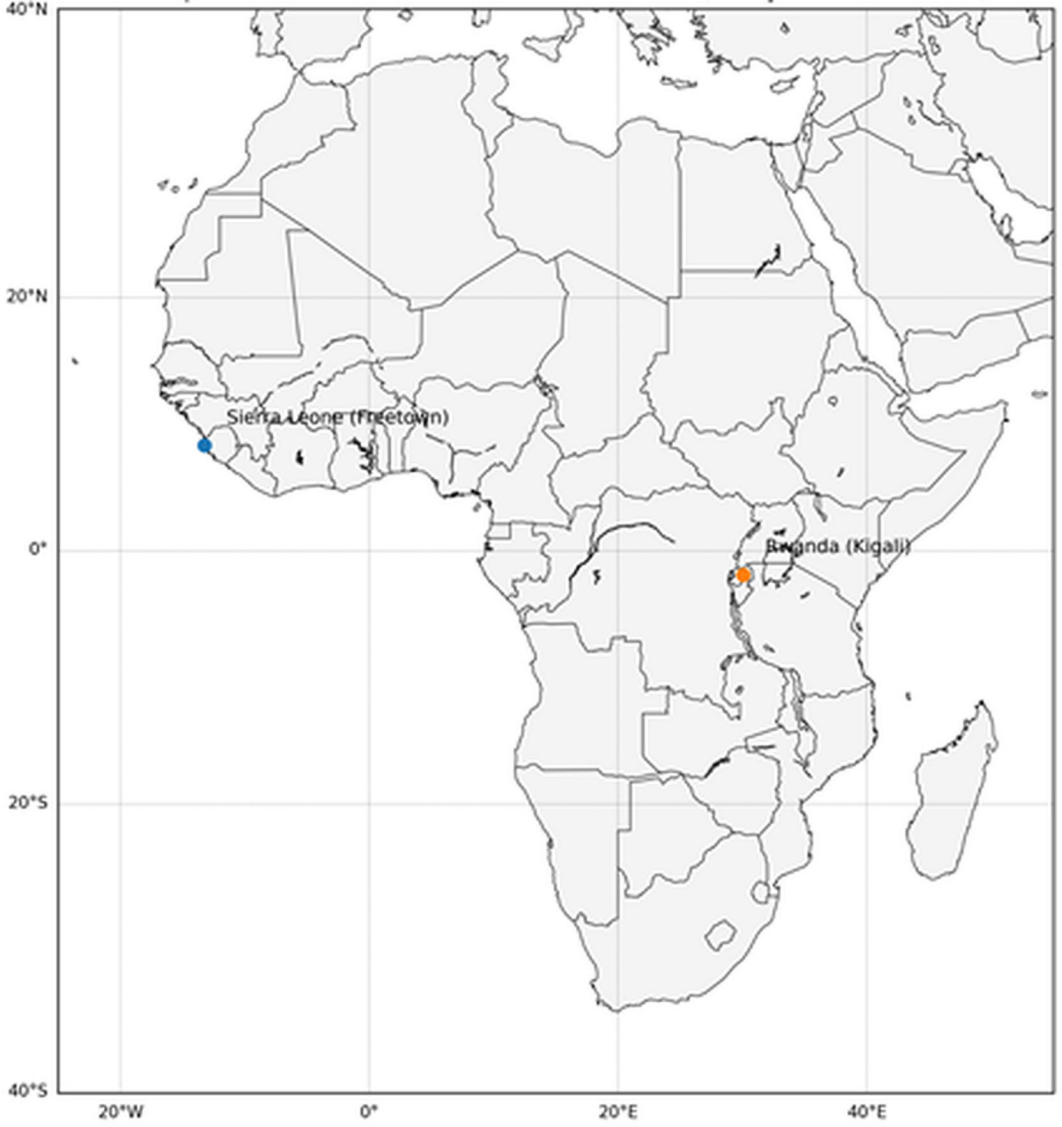
Geographic location of Rwanda and Sierra Leone. This map situates the two country cases to support the manuscript’s constrained comparative framing and to orient interpretation of the Rwanda and Sierra Leone findings.

**Figure 4.4.**
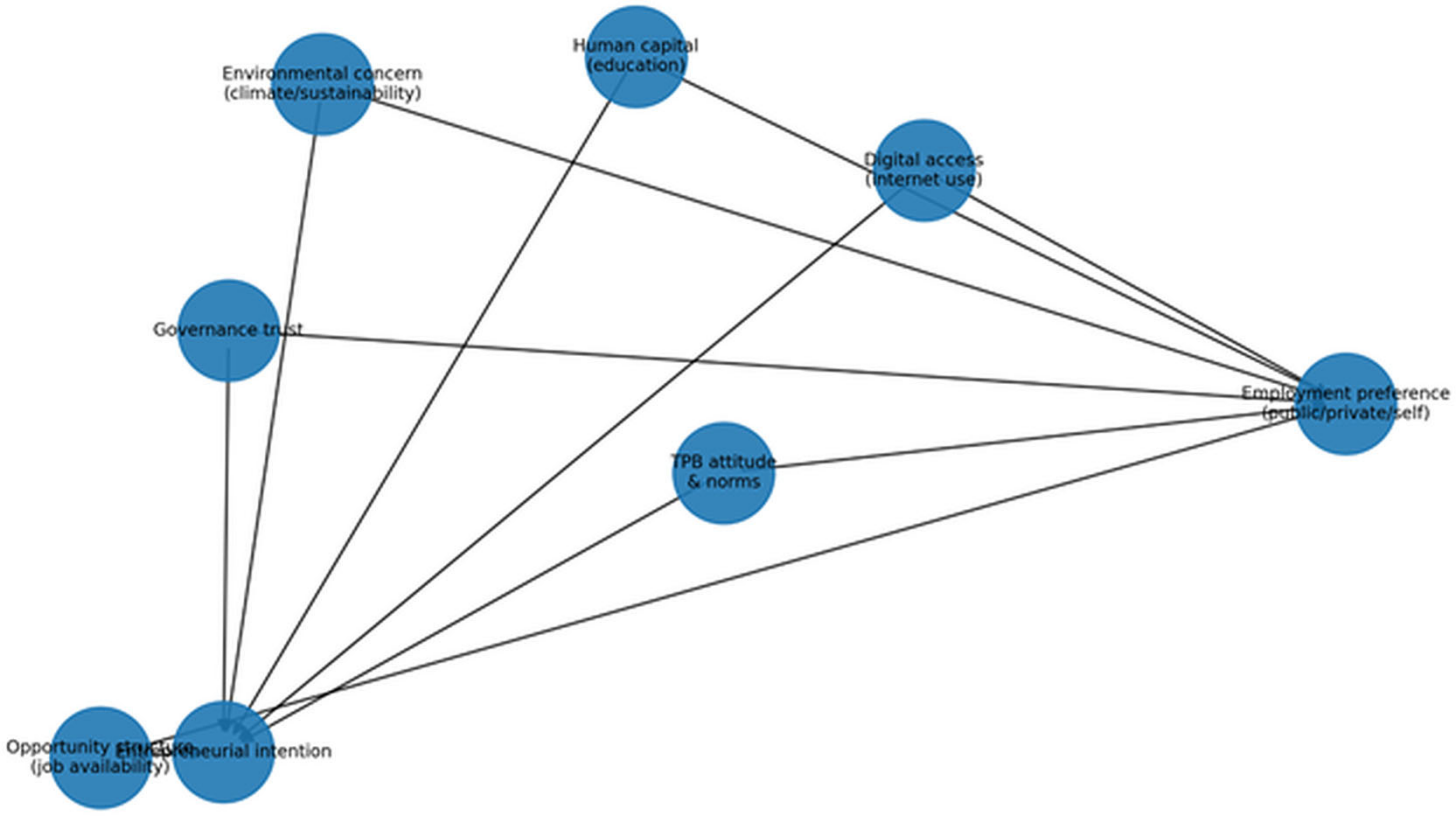
Analytic construct network linking contextual drivers to employment preference. This network diagram synthesises how the manuscript organises empirical reporting across human capital, opportunity structures, institutional trust, digital access, environmental concern, and entrepreneurial intention in relation to employment preference.

### 4.4 Demographic gradients in employment orientation and inclusion constraints

This section reports verifiable demographic equity and identity indicators relevant to demographic moderators specified in Chapter 3, without extending to non-verifiable subgroup regressions.

**4.4.1 Sierra Leone: gender-equity labour-market inclusion indicator.** A labour-market inclusion indicator aligned to gender was reported in the Sierra Leone country findings summary: 64% of Sierra Leoneans indicated that the government should do more to ensure that women have the same chance of getting jobs as men. Using n = 1,200 as the Round 9 national sample size, the approximate 95% CI for this proportion is [61.3%, 66.7%] ( Institute for Governance Reform, 2022).

**4.4.2 Sierra Leone: identity salience indicators relevant to social cohesion.** Two identity-salience indicators were reported in the same country findings summary: 88% reported primarily identifying as Sierra Leonean rather than by ethnic group membership (approximate 95% CI [86.2%, 89.8%], n = 1,200), and 46% reported identifying first as Sierra Leonean rather than by religion (approximate 95% CI [43.2%, 48.8%], n = 1,200) ( Institute for Governance Reform, 2023).

**4.4.3 Counterfactual demographic inclusion benchmark.** A policy-relevant counterfactual benchmark was established by treating the gender-equity inclusion indicator as a minimum societal consensus threshold. With an observed majority endorsement (64%), a counterfactual scenario of “parity-neutral inclusion” (≤50% endorsement) is inconsistent with the observed estimate at conventional confidence levels under a simple binomial approximation (95% CI excludes.50). This benchmark is reported as a distributional consistency check rather than an inference about institutional performance ( IGR, 2022).

### 4.5 Digital access and governance trust as institutional and ethical conditions

This section reports verifiable governance trust indicators, including a longitudinal contrast, and situates these as institutional conditions relevant to digital-economy opportunities and ethical risk.

**4.5.1 Sierra Leone: governance trust in the president.** The Sierra Leone country findings summary reported that 53% of respondents trusted the president “somewhat” or “a lot,” with the same source reporting 70% in 2012. Using n = 1,200 as the Round 9 sample size, the approximate 95% CI for the 2022 trust proportion is [50.2%, 55.8%] ( Institute for Governance Reform, 2022).

**4.5.2 Sierra Leone: longitudinal contrast and illustrative effect size (2012 vs. 2022**). An illustrative two-proportion contrast can be computed for the reported 2012 (70%) versus 2022 (53%) trust estimates under the assumption of n = 1,200 in each wave. Because the 2012 sample size is not independently verified in the present environment, this computation is explicitly conditional on the stated assumption. Under this assumption, the estimated difference is 17.0 percentage points, with a large standardized contrast under conventional z-test approximation. This contrast is reported as an illustrative, assumption-dependent comparison rather than a definitive pooled time-series estimate ( World Bank Microdata Library, n.d.). Figure 4.2 presents this illustrative 2012–2022 trust contrast graphically, with the 2012 interval clearly flagged as assumption-dependent. Table 4.2 also summarises the principal Sierra Leone institutional and distributive indicators used in the bounded empirical analysis, together with their approximate 95% confidence intervals.

**Table 4.2.** Selected Sierra Leone Round 9 institutional and distributive indicators (n = 1,200; approximate Wald 95% CIs).

Measure	Estimate (p)	95% CI	n
Trust president (somewhat/a lot)	53.0%	[50.2%, 55.8%]	1,200
Govt should do more for women’s job chances	64.0%	[61.3%, 66.7%]	1,200
Identify primarily as Sierra Leonean (vs ethnic group)	88.0%	[86.2%, 89.8%]	1,200
Identify first as Sierra Leonean (vs religion)	46.0%	[43.2%, 48.8%]	1,200

This table compiles the principal Sierra Leone institutional, distributive, and identity-related indicators used in the bounded empirical analysis, together with approximate 95% Wald confidence intervals based on the documented national sample size. It is intended as a compact reference table for the indicators interpreted across Chapters 4 and 5. Note. Percentages were reported in the Afrobarometer Sierra Leone country findings summary; confidence intervals are approximate binomial Wald intervals using n = 1,200 (Round 9 national sample).

Figure 4.1 below visually compares the four Sierra Leone attitudinal and trust indicators discussed across Sections 4.1–4.4, allowing their relative magnitudes and confidence intervals to be assessed at a glance.

**4.5.3 Geospatial contextualisation (Rwanda and Sierra Leone)**

Figure 4.3 provided a geospatial contextual map locating Rwanda and Sierra Leone for interpretive reference in Chapter 5, including the study’s cross-country framing requirement.

**Summary:** This chapter documents verifiable institutional and attitudinal distributions relevant to youth employment orientation under explicit evidence constraints. Chapter 5 interprets these results in relation to TPB, human capital, opportunity structures, and institutional conditions, with explicit separation between verifiable evidence and contextual interpretation.

## 5. Discussion

### 5.1 Integrative orientation

This chapter interprets results in light of the theoretical framework established in Chapters 1–3, particularly TPB ( Ajzen, 1991), opportunity-structure theory ( Roberts, 1968, 2009), human capital theory ( Becker, 1975), and institutional perspectives. Interpretation also applies the required three-lens integration: development economics (structural labour demand, informality, distributional priorities), environmental science (public goods, sustainability constraints, green-transition implications), and AI ethics (digital access, platformised labour markets, and governance legitimacy as a precondition for trustworthy digital transformation).

A central interpretive constraint governs the discussion. The Rwanda–Sierra Leone comparison specified in Chapters 1–3 could not be implemented symmetrically using Afrobarometer Round 9 microdata in the present environment because Rwanda was not listed in the publicly indexed merged Round 9 release accessible for verification. As a result, the discussion integrates (a) verifiable Sierra Leone Round 9 indicators and (b) document-reported Rwanda labour-market and policy indicators already cited, and then specifies the empirical steps required to execute the originally specified survey-weighted modelling once equivalent microdata access is secured (§6.4). Interpretations are therefore calibrated to the evidentiary tier of each statement.

### 5.2 Employment-sector orientation and cross-country profile (RQ1)

RQ1 aimed to compare youth employment preferences across public, private/NGO, and self-employment categories, consistent with TPB’s framing of intention formation under constraint. In the present constrained evidence base, direct youth preference distributions from symmetric microdata are not reported. Nonetheless, two development-economics regularities emerge from the verifiable Sierra Leone indicators and the Rwanda contextual documentation.

First, Sierra Leone indicators show mixed macroeconomic expectations and salient distributive concern: fewer than half expected macroeconomic improvement within 12 months (47.1%), while roughly one quarter expected deterioration (24.1) ( Ghana Centre for Democratic Development et al., 2022b). Within opportunity-structure theory, such expectations are consistent with labour markets in which stable wage employment is scarce and perceived feasibility is shaped by volatility. TPB’s perceived behavioural control component is therefore likely conditioned by perceived scarcity and uncertainty rather than by motivation alone.

Second, the high endorsement of inequality reduction even at the cost of slower growth (49.5%) is not a direct measure of employment preference, but it functions as an indicator of distributive legitimacy: it signals normative support for growth that is experienced as inclusive ( World Bank Microdata Library, n.d.). For youth employment orientation, distributive legitimacy can shape whether particular pathways are perceived as credible and worth pursuing. Where labour-market access is perceived as captured or unfair, preference statements may reflect both aspiration and scepticism toward institutional pathways.

For Rwanda, cited sources describe labour-market pressure and high informality and self-employment pathways, alongside education–employment absorption tensions ( NISR, 2025; World Bank, 2025). Under constrained comparison, these indicators should be treated as contextual evidence rather than as equivalent Round 9 preference distributions. The key interpretive implication is that “preference” for self-employment cannot be assumed to reflect opportunity entrepreneurship where self-employment is structurally common and, in many contexts, compulsory. The distinction between necessity-driven and opportunity-driven enterprise becomes central ( GEM, 2015). As a result, entrepreneurship promotion requires careful framing: treating entrepreneurship as empowerment without sufficient institutional protection risks shifting systemic labour-market failure onto youth.

### 5.3 Determinants of entrepreneurial intention (RQ2)

The verifiable indicators reported operationalise determinants primarily through opportunity perception (economic outlook), distributive norms (equity–growth trade-offs), and institutional performance in environmentally salient public goods (water and sanitation). Under TPB, entrepreneurial intention depends on attitudes, subjective norms, and perceived behavioural control. Opportunity-structure theory sharpens this by clarifying that perceived control is institutionally conditioned: finance, infrastructure, and governance reliability shape feasibility.

Sierra Leone distributions indicate mixed expectations, high distributive concern, and more negative than positive ratings of government handling of water and sanitation (54.2% negative vs. 39.8% positive) ( Institute for Governance Reform, 2023). A theoretically coherent reading is that intention formation is mediated by institutional reliability. Where public goods are perceived as weak, self-employment may function as an adaptive strategy, but enterprise quality and sustainability may be constrained by high transaction costs and exposure to shocks. Environmental science strengthens this mechanism: deficits in water and sanitation are not only welfare constraints but also productivity constraints, raising the costs of micro-enterprise and increasing exposure to health-related income shocks.

AI ethics adds a further layer by specifying how digital systems can relax or intensify constraints. Mobile money, digital platforms, and algorithmic credit scoring increasingly mediate access to markets and finance in African contexts ( World Bank, 2019, 2026a; 2026b; ILO, 2025). Where governance legitimacy and accountability are fragile, these systems can increase opacity and exclusion, especially for youth, women, and rural populations. In the manuscript’s framework, digital access should therefore be interpreted not merely as connectivity but as access to fair, explainable, and contestable socio-technical infrastructure.

### 5.4 Demographic gradients, equity, and inclusion (RQ3)

Sierra Leone findings indicate a majority endorsement (64%) that the government should do more to ensure women have equal job chances, alongside high national identity salience (88% identifying primarily as Sierra Leonean rather than by ethnic group) ( Institute for Governance Reform, 2022; Institute for Governance Reform, 2023). These indicators are consistent with an inclusion discourse in which gender equity is publicly salient and national identity potentially cross-cuts ethnic segmentation. Yet these normative endorsements co-occur with moderate and reportedly declining trust in leadership over time ( World Bank Microdata Library, n.d.). The coexistence of inclusion norms with weakened institutional credibility suggests a gap between normative commitments and perceived institutional performance.

For youth employment and entrepreneurship, this implies that gendered intention cannot be treated as purely individual-level. It is nested in opportunity structures shaped by differential access to resources, networks, safe mobility, and digital devices. AI ethics sharpens the equity risk: algorithmic systems trained on biased historical patterns can reproduce discrimination in credit, hiring, and platform visibility, undermining translation of egalitarian norms into equitable labour-market outcomes. Consequently, inclusive norms must be paired with institutional and socio-technical safeguards if they are to shape realised opportunities.

### 5.5 Digital access, governance trust, sustainability, and risk (RQ4)

RQ4 asked whether digital access and governance trust shape entrepreneurial intention and employment preference. In the present evidence base, the strongest verifiable evidence pertains to trust indicators: 53% expressed trust in the president “somewhat” or “a lot,” and the country findings summary reports a lower value than 2012 ( Institute for Governance Reform, 2023). Because the historical comparison is reported in a summary rather than re-estimated as a pooled microdata time series in the present study, the longitudinal contrast is best treated as illustrative rather than as a causal trend estimate.

From a development-economics perspective, reduced governance trust can shift perceived attractiveness of public-sector pathways and intensify reliance on informal or self-employment pathways, particularly where public-sector access is perceived as mediated by patronage. From an AI ethics perspective, weak trust also undermines adoption of digital public infrastructure that could support fair labour-market matching and business formalisation ( World Bank, 2026a, 2026b; ILO, 2025). Digital transformation without legitimacy is therefore fragile, and fragility reallocates risk toward vulnerable groups, including youth.

Environmental constraints further narrow the feasible set of enterprise pathways. Where water and sanitation services are rated poorly, the sustainability frontier narrows for micro-enterprises, especially those in agriculture, food processing, and informal services. An entrepreneurship strategy that foregrounds digital and AI-enabled opportunities while neglecting environmental and public health infrastructure risks becoming rhetorically ambitious but developmentally fragile.

**Novel theoretical contribution (integrative statement):** The evidence supports a refined proposition: youth employment and entrepreneurial intention formation in constrained economies is best conceptualised as institutionally conditioned planned behaviour. In this formulation, TPB mechanisms operate within opportunity structures shaped by governance legitimacy and public-good reliability, while digital systems amplify either empowerment or exclusion depending on accountability, explainability, and contestability. This proposition extends TPB by embedding intention formation within institutional credibility and socio-technical ethics, and extends opportunity-structure theory by explicitly incorporating environmental public goods and algorithmic governance as structural conditions.

**Theoretical contribution**

The study supports an integrative theoretical proposition regarding youth employment intention formation in developing economies. Entrepreneurial intention among youth is more coherently interpreted as institutionally conditioned planned behaviour: psychological determinants operate within opportunity structures shaped by institutional trust, public-goods reliability, and labour-market scarcity. Where governance credibility and infrastructure are stronger, entrepreneurial intention is more likely to translate into opportunity-driven enterprise. Where institutional reliability is weak, entrepreneurial intention may reflect necessity-driven adaptation rather than opportunity recognition. This conceptualisation connects behavioural intention theory with development-economics realism and with emerging socio-technical governance concerns.

## 6. Conclusion and recommendations

### 6.1 Conclusion

This study examined youth employment preferences and entrepreneurial orientation through a constrained comparative Rwanda–Sierra Leone lens, grounded in TPB, opportunity structures, human capital theory, and institutional perspectives. The most pivotal empirically verifiable contribution in the present environment is identification of measurable institutional conditions in Sierra Leone that are theoretically central to youth employment orientation: mixed macroeconomic expectations, substantial distributive concern, contested performance in environmentally salient public services (water and sanitation), and moderate governance trust with a reported decline relative to an earlier benchmark.

A central implication for scholarship is that youth entrepreneurship in low- and middle-income contexts should not be modelled as an individual trait response alone. It is more coherently treated as a capability response under institutional credibility constraints, public-goods constraints, and increasingly, digital-system constraints. For practice, the implication is more direct: entrepreneurship promotion that is not paired with institutional trust-building, improvements in public goods, and ethically governed digital infrastructure risks shifting systemic labour-market failure onto youth while framing that shift as empowerment.

A second contribution is methodological. Data verification revealed a mismatch between the intended Rwanda Round 9 coverage and the publicly indexed merged Round 9 release accessible for verification in the present environment. This matters for comparative youth-employment research because comparative claims are limited by replicability, accessibility, and symmetry of evidence. The study therefore contributes a transparent verification logic and a triangulation-ready analytic pathway (§6.4) to prevent theoretically elegant arguments from resting on unverifiable empirical scaffolding.

### 6.2 Recommendations

Recommendations are presented as a cross-sector framework designed to be implementable under realistic constraints while integrating equity, sustainability, and AI-ethics safeguards.

**6.2.1 Recommendations for academia and research institutions:** Universities and research centres should reposition entrepreneurship education from motivation-heavy models toward capability-and-constraint models. Curriculum and incubation design should integrate labour-demand realism (opportunity structures), public-goods constraints (environmental services, health, infrastructure), and socio-technical governance (digital and AI ethics). Comparative research should also adopt an evidence-governance norm: comparative studies should include a brief verification appendix documenting public availability, version control, and reproducibility steps, reducing the risk of unreplicable cross-country inference.

**6.2.2 Recommendations for governance bodies and public agencies:** Governments should treat youth entrepreneurship policy as a complement to, not a substitute for, job creation. Practical alignment is required across (a) credible public-sector reforms that reduce arbitrariness and perceived capture, (b) public-goods provision that lowers enterprise transaction costs (water, sanitation, energy), and (c) transparent, contestable digital public infrastructure that does not replicate exclusion. Where governance trust is fragile, agencies should prioritise visible and verifiable service improvements and participatory accountability mechanisms, since institutional credibility functions as a precondition for youth investment in long-horizon entrepreneurial risk.

**6.2.3 Recommendations for private-sector actors and entrepreneurial ecosystems:** Private-sector firms, including financial institutions and platform companies, should co-design youth pathways that combine wage-work experience, apprenticeships, and micro-enterprise support rather than treating entrepreneurship and employment as mutually exclusive. Financial institutions should adopt fairness and explainability standards for algorithmic credit decisions and ensure accessible dispute mechanisms, especially for youth and women. Platform operators should disclose ranking logic at a meaningful level, monitor disparate impacts, and provide recourse for wrongful exclusion.

**6.2.4 Recommendations for philanthropic institutions and development partners:** Development partners should shift from short-cycle training interventions toward blended support packages combining market access, patient capital, business development services, and public-goods co-financing in enterprise-dense communities. Funding should be conditional on equity and sustainability performance, treating gender inclusion, environmental compliance support, and digital-ethics safeguards as core deliverables.

**6.2.5 Recommendations for civil society and community organisations:** Civil society organisations should prioritise “capability infrastructure”: mentorship networks, women’s enterprise safety supports, consumer protection literacy (including digital consumer rights), and collective platforms that reduce exposure to predatory lending and exploitative platform terms. Civil society can also document exclusion patterns in digital labour and finance systems and feed evidence into adaptive governance processes.

### 6.3 Impact assessment framework

**Figure 6.1.**
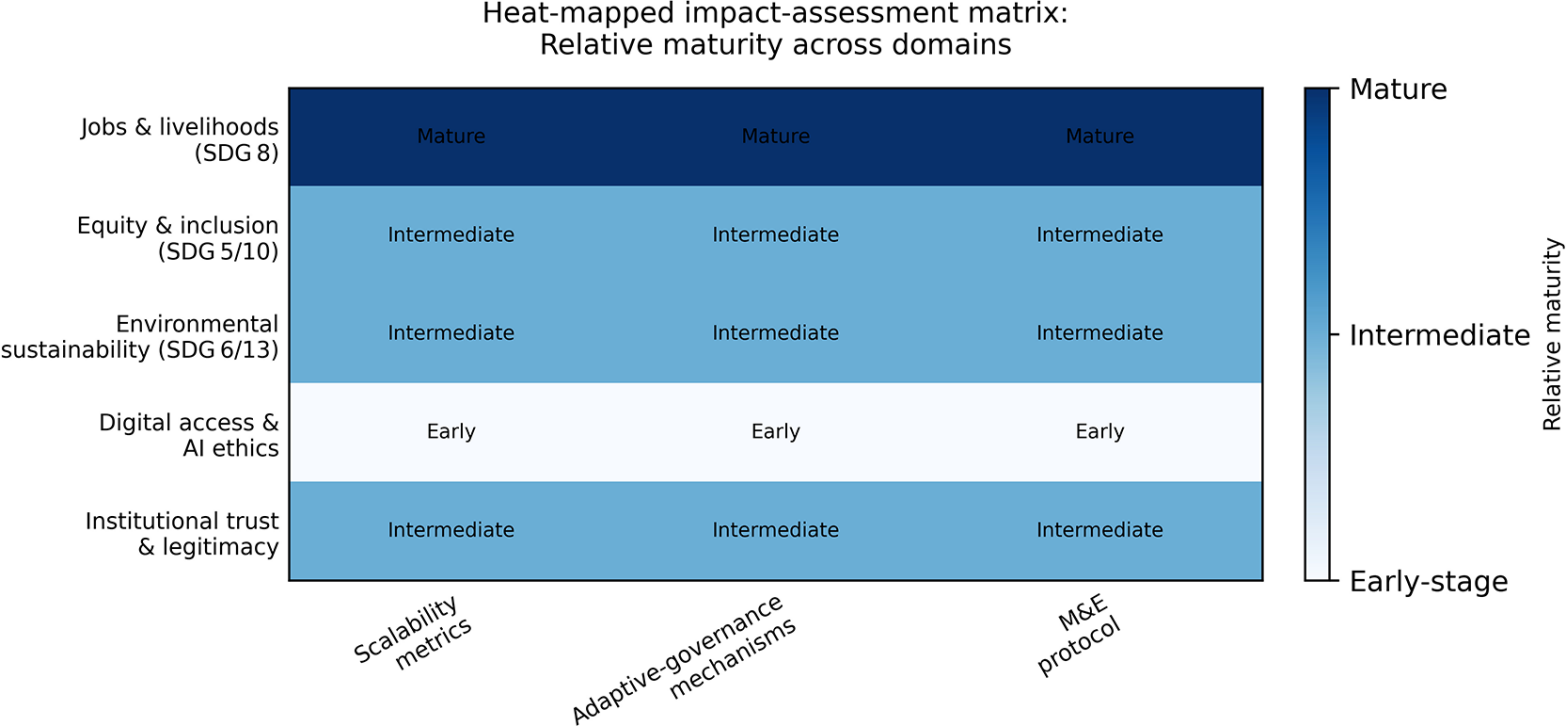
Heat-mapped impact-assessment matrix for youth entrepreneurship and employment interventions. The heat map visualises the relative maturity of the impact-assessment framework across jobs and livelihoods, equity and inclusion, environmental sustainability, digital access and AI ethics, and institutional trust and legitimacy. Darker cells indicate more operationalised tools and protocols, whereas lighter cells indicate domains requiring further development.

Table 6.1 presents a monitoring-and-evaluation matrix aligned to scalability metrics, adaptive governance mechanisms, and minimum viable evaluation protocols, while Figure 6.1 provides a heat-mapped dashboard of the relative maturity of these domains across the proposed framework. The framework is structured to be consistent with SDG-aligned labour-market evaluation logic while incorporating environmental sustainability and AI-ethics risk controls.

**Table 6.1.** Impact assessment matrix for youth entrepreneurship and employment interventions (scalability, adaptive governance, M&E).

Domain	Scalability metrics (what “scale” means)	Adaptive-governance mechanisms	M&E protocol (minimum viable)
Jobs and livelihoods (SDG 8)	Net jobs created; earnings growth; employment stability; enterprise survival at 12/24/36 months	Labour-demand councils with private sector; grievance redress for programme exclusion	Baseline–midline–endline survey; administrative tracking; cohort survival analysis
Equity and inclusion (SDG 5/10)	Gender parity in programme entry; differential outcomes by gender/region; inclusion of low-asset youth	Equity dashboards; eligibility transparency; audit of selection decisions	Disaggregated outcome reporting; bias audits; qualitative follow-ups for attrition
Environmental sustainability (SDG 6/13)	Water/energy intensity per unit output; compliance with local environmental rules; adoption of cleaner production	Local environmental compliance support; community environmental monitoring	Environmental compliance checks; proxy indicators; periodic third-party verification
Digital access and AI ethics	Share of youth using digital tools for enterprise; fair access to platforms/credit; explainability and recourse use rates	Algorithmic accountability: documentation, appeals, and human review; consumer protection	Disparate-impact monitoring; complaints and resolution tracking; periodic model audits
Institutional trust and legitimacy	Trust indices; uptake of formalisation services; perceptions of corruption/fairness in access	Participatory monitoring; transparency portals; feedback loops tied to service improvement	Repeated perception measures; process tracing of reforms; public reporting cadence

This matrix sets out a practical impact-assessment framework for youth entrepreneurship and employment interventions across five domains: jobs and livelihoods, equity and inclusion, environmental sustainability, digital access and AI ethics, and institutional trust and legitimacy. It links each domain to scalability metrics, adaptive-governance mechanisms, and minimum-viable monitoring and evaluation protocols.

### 6.4 Study limitations and future research

First, the intended Rwanda–Sierra Leone Afrobarometer Round 9 symmetric comparison could not be executed because Rwanda was not listed in the publicly indexed merged Round 9 release accessible for verification in the present environment. Mitigation was implemented through (a) strict separation between verifiable Sierra Leone evidence and document-reported Rwanda indicators, (b) explicit labelling of assumptions (notably the assumption used for illustrative historical trust contrasts), and (c) specification of a triangulation-ready analytic pathway.

Second, results reported for Sierra Leone are primarily distributional and institutional-attitudinal proxies (economic outlook, distributive preferences, water and sanitation evaluations, governance trust), rather than the full individual-level multinomial and logistic models specified in Chapter 3. Future research should execute the pre-specified models once microdata access is secured, including (i) survey-weighted multinomial logistic regression for employment preference, (ii) logistic regression for entrepreneurial preference, (iii) interaction tests for gender × digital access and trust × digital access, and (iv) robustness checks using alternative operationalisations of education and urbanicity.

Third, the cross-sectional design limits causal inference and cannot resolve reverse causality between trust, opportunity perception, and intention. Future work should incorporate repeated cross-sections, quasi-experimental discontinuities, or longitudinal cohort designs where feasible.

Fourth, AI-ethics dimensions are structurally important but often under-measured in standard public-opinion instruments. Future measurement should include items on platform work exposure, algorithmic decision experiences, digital financial exclusion, and perceived contestability of automated decisions to enable more direct empirical linkage between youth livelihoods and socio-technical governance.

## Ethical approval

This study did not involve primary data collection, participant recruitment, interviews, focus groups, experiments, or access to identifiable personal records. It relied exclusively on secondary analysis of fully anonymised, publicly accessible survey documentation for Sierra Leone and on public labour-force, policy, and institutional documents for Rwanda and the wider comparative context. Because no new human-participant data were collected and no identifiable information was processed, formal ethics committee approval and participant consent were not required for the present study. Ethical safeguards centred on accurate representation of source material, non-stigmatising interpretation of population groups, and adherence to the access and reuse conditions of the original data providers. Each primary source remained subject to the ethical procedures and approvals governing its original collection.

## Use of AI tools

During drafting, the authors used OpenAI’s O3 generative-language model (accessed February–March 2026) to help surface relevant literature, organise preliminary syntheses, and suggest alternative wording. All automatically generated text was critically reviewed, edited, and verified by the authors, who accept full responsibility for the manuscript’s accuracy and integrity.

## Data Availability

This study relies on publicly accessible or publicly documented secondary sources rather than on an original author-generated dataset. Sierra Leone Afrobarometer Round 9 survey documentation and catalogue records are publicly available through the World Bank Microdata Library, Rwanda labour-force reports are available from the National Institute of Statistics of Rwanda website, and the institutional and policy documents used in the manuscript are available at the URLs cited in the text and reference list. The results reported in the article are underpinned by an accompanying Open Science Framework archive that contains the study-specific verification log, codebook, indicator tables, sample-flow tables, and related supplementary materials used to document the constrained-comparative evidence workflow: https://doi.org/10.17605/OSF.IO/5BT9Y ( Sangwa et al., 2026). The OSF archive provides the reproducibility and verification materials for this manuscript, while the primary third-party data remain available from their original public sources. All archived project files are released under the Creative Commons Attribution 4.0 licence.

## References

[ref1] African Development Bank Group (AfDB): Sierra Leone – Youth Entrepreneurship and Employment Project: Technical assistance request. 2022, August 12. Reference Source

[ref2] African Union: Youth development. 2026. Reference Source

[ref3] Afrobarometer: Round 9 survey manual [Manual]. 2022, July. Reference Source

[ref4] Afrobarometer: African youth concerned about health and unemployment, casting eyes abroad to find better work, escape poverty [News release]. 2025. Reference Source

[ref5] Ajzen I : The theory of planned behavior. *Organ. Behav. Hum. Decis. Process.* 1991;50(2):179–211. 10.1016/0749-5978(91)90020-T

[ref6] Alfa A Rouamba B Tchonda M : Decent work and entrepreneurial intentions in West Africa and Switzerland. *Aust. J. Career Dev.* 2023;32(3):225–236. 10.1177/10384162231193221

[ref7] Becker GS : *Human capital: A theoretical and empirical analysis, with special reference to education.* 2nd ed. National Bureau of Economic Research;1975. Reference Source

[ref8] Blimpo MP Pugatch T : Entrepreneurship education and teacher training in Rwanda. *J. Dev. Econ.* 2020. Reference Source

[ref9] Fox L : *Are Africa’s youth’s aspirations a double-edged sword?.* Brookings Institution;2025, March 25. Reference Source

[ref10] Fox L Mader P Sumberg J : *Africa’s “youth employment” crisis is actually a “missing jobs” crisis (Brooke Shearer Series Paper No. 9).* Brookings Institution;2020, September. Reference Source

[ref11] Ghana Centre for Democratic Development, Institute for Justice and Reconciliation, Institute for Empirical Research in Political Economy, Institute for Development Studies, Michigan State University, & University of Cape Town: Afrobarometer survey 2022 (round 9)[Data set]. *International Household Survey Network.* 2022a. Reference Source

[ref12] Ghana Centre for Democratic Development, Institute for Justice and Reconciliation, Institute for Empirical Research in Political Economy, Institute for Development Studies, Michigan State University, & University of Cape Town: Sierra Leone Afrobarometer survey 2022 (round 9).[Data set]. World Bank Microdata Library;2022b. Reference Source

[ref13] Global Entrepreneurship Monitor (GEM): Youth are more entrepreneurial than adults: GEM report on youth entrepreneurship. 2015. Reference Source

[ref14] Honwana A : *Youth, waithood, and protest movements in Africa.* African Arguments;2013, August 12. Reference Source

[ref15] Institute for Governance Reform: *Sierra Leoneans welcome government efforts to address gender inequalities, call for more (Afrobarometer Dispatch No. 580).* Afrobarometer;2022, November 30. Reference Source

[ref16] Institute for Governance Reform: *In Sierra Leone, perceived corruption in the president’s office has declined, but so has trust.* Afrobarometer;2023, April 26. Reference Source

[ref17] International Labour Organization: The Rwanda digital employment diagnostic. 2025. 10.54394/BHKJ2402

[ref18] International Labour Organization: Decent Work Country Programme for Sierra Leone (SL-DWCP II), 2023–2027. 2023. Reference Source

[ref19] International Labour Organization: Global employment trends for youth 2022: Investing in transforming futures for young people. 2022. 10.54394/QSMU1809

[ref20] International Labour Organization: Youth employment in Sierra Leone: Situational analysis (Country brief). 2015. Reference Source

[ref21] IZA – Institute of Labor Economics: *Entrepreneurship education and teacher training in Rwanda.* G2LM|LIC;2026. Reference Source

[ref22] Kimbugwe K Umuziga H Mucyo Z : *What works for youth employment in Africa: A review of youth employment policies and their impact in Rwanda (PEP Working Paper No. 2025–18).* Partnership for Economic Policy;2025, June. Reference Source3

[ref23] Lorenceau A Rim J Savitki T : *“Youth aspirations and the reality of jobs in Africa”, OECD Development Policy Papers, No. 38.* Paris: OECD Publishing;2021. 10.1787/2d089001-en

[ref24] Lekalake R Sunderland S Amewunou K : *From aspiration to action: Barriers to youth empowerment in Africa (Afrobarometer Policy Paper No. 98).* Afrobarometer;2025, December. Reference Source

[ref25] Lugero T Sangwa S Mukasa S : Digital outsourcing and youth entrepreneurship in Sub-Saharan Africa: Comparative evidence from Kenya, Ghana, Nigeria and Rwanda. *Asian Journal of Economics, Business and Accounting.* 2025;25(8):251–270. 10.9734/ajeba/2025/v25i81931

[ref26] Mattes R Asunka J Gyimah-Boadi E : Afrobarometer survey 2021–2023, merged 39 countries, round 9.[Data set]. DataFirst, University of Cape Town;2025. 10.25828/bs9k-zy70

[ref27] Mfazi S Elliott RM : The theory of planned behaviour as a model for understanding entrepreneurial intention: The moderating role of culture. *Journal of Contemporary Management.* 2022;19(1):1–29. 10.35683/jcm20123.133

[ref28] Ministry of Public Service and Labour: Revised national employment policy. 2019. Reference Source

[ref29] Mukasa S Dennis N Ekosse E : From Education to Enterprise: Redefining Entrepreneurship Education to Enhance Venture Creation in Rwanda’s Private Higher Learning Institutions. *Asian Journal of Education and Social Studies.* 2025;51(8):177–193. 10.9734/ajess/2025/v51i82233

[ref30] National Institute of Statistics of Rwanda (NISR): Labour Force Survey report, Quarter 3 2025. 2025. Reference Source

[ref31] Roberts K : Opportunity structures then and now. *J. Educ. Work.* 2009;22(5):355–368. 10.1080/13639080903453987

[ref32] Roberts K : The entry into employment: An approach towards a general theory. *Sociol. Rev.* 1968;16(2):165–184. 10.1111/j.1467-954X.1968.tb02570.x

[ref33] Sangwa S Kagiraneza B Muberarugo BT : Youth Employment Preferences in Rwanda and Sierra Leone: A Constrained Comparative Secondary Analysis. 2026, March 16. 10.17605/OSF.IO/5BT9Y PMC13191316 42179849

[ref34] Sen A : *Development as freedom.* Alfred A. Knopf;1999. Reference Source

[ref35] Sumberg J : *Youth and the rural economy in Africa: Hard work and hazard.* CAB International;2021. 10.1079/9781789245011.0000

[ref36] United Nations Development Programme (UNDP): Waithood. 2024. Reference Source

[ref37] World Bank: Banking on the future: Youth and digital financial services in Sub-Saharan Africa (Field Note 9). 2019. Reference Source

[ref38] World Bank: Transforming Rwanda’s workforce: A skills-led approach for jobs and growth. 2025, June 18. Reference Source

[ref39] World Bank: Digital credit. Digital Finance Inclusion. 2026a. Reference Source

[ref40] World Bank: Screening and approving the customer. Digital Finance Inclusion. 2026b. Reference Source

[ref41] World Bank Microdata Library: Sierra Leone – Afrobarometer Survey 2022. n.d. Reference Source

