# Supplementary material for: Youth Employment Preferences in Rwanda and Sierra Leone: A Constrained Comparative Secondary Analysis
Source: Open Res Afr. 2026 Apr 14;9:14. [Version 1] doi: 10.12688/openresafrica.16530.1 (PMC13191316; doi:10.12688/openresafrica.16530.1)
Supplement: Peer review history

# Reviewer report: Reviewer response for version 1

**DOI:** 10.21956/openresafrica.17718.r34614

**Published:** 2026-05-20

**Authors:** Md Jahangir Alam

**Affiliations:** 1 University of Dhaka, Dhaka, Bangladesh

1. The absence of Rwanda microdata weakens comparative validity, making conclusions largely contextual rather than empirically balanced across both national cases.
2. Heavy reliance on documentary sources reduces analytical originality and limits the robustness of empirical findings for comparative statistical interpretation.
3. Cross-sectional design prevents identifying causal relationships between institutional trust, opportunity structures, and entrepreneurial preferences among youth populations.
4. Several constructs, like digital access and institutional trust, are insufficiently operationalized, affecting clarity and measurement precision.
5. Combining multiple theories creates conceptual richness but reduces coherence, making analytical integration occasionally appear forced rather than naturally aligned.
6. No triangulation through interviews, focus groups, or expert consultation reduces contextual depth and interpretive reliability.
7. Promised multinomial regression analysis remains unexecuted, significantly limiting explanatory power and inferential contribution.
8. Findings are context-specific and cannot be generalized across Sub-Saharan Africa due to evidence asymmetry and sample limitations.
9. The review emphasizes African contexts but overlooks broader comparative insights from Asian countries, particularly Japan’s youth employment transition models.
10. Policy recommendations remain broad and would benefit from clearer implementation strategies, measurable indicators, and institutional accountability mechanisms.
11. Also, I recommend reading some related literature that might strengthen your findings. (Quality assurance of science and technology universities in Bangladesh: a catalyst for sustainable employment during the 4IR; The role of higher education for sustainable employment in Bangladesh; Sustainable employment for vocational education and training graduates: the case of future skills matching in Bangladesh)

Is the work clearly and accurately presented and does it cite the current literature?

Yes

If applicable, is the statistical analysis and its interpretation appropriate?

Yes

Are all the source data underlying the results available to ensure full reproducibility?

Yes

Is the study design appropriate and is the work technically sound?

Yes

Are the conclusions drawn adequately supported by the results?

Yes

Are sufficient details of methods and analysis provided to allow replication by others?

Yes

Reviewer Expertise:

Employability

I confirm that I have read this submission and believe that I have an appropriate level of expertise to confirm that it is of an acceptable scientific standard.

# Reviewer report: Reviewer response for version 1

**DOI:** 10.21956/openresafrica.17718.r34437

**Published:** 2026-05-19

**Authors:** Thandukwazi Richman Ncube

**Affiliations:** 1 Durban University of Technology, Durban, South Africa

**Overall Summary**

 The paper addresses a critical issue in African development: the tension between youth aspirations for formal work and the structural reality of informal self-employment. The authors employ a constrained comparative design necessitated by the unavailability of Rwandan microdata, shifting the focus to a bounded-evidence strategy. The paper is theoretically robust, integrating the Theory of Planned Behaviours with Opportunity Structure Theory. However, its primary challenge lies in the evidentiary asymmetry between the two case studies.

**Strengths**

**1. Methodological Transparency and Integrity**

 The authors deserve significant credit for their transparency. Rather than massaging the data or ignoring the missing Rwandan microdata, they explicitly define a constrained comparative approach. This honesty prevents false equivalence and sets a high standard for open-science reporting.

**2. Theoretical Innovation**

 The introduction of the “Institutionally Conditioned Planned-Behaviour” (ICPB) framework is a notable contribution. By nesting the Theory of Planned Behaviours (individual intention) within Opportunity Structure Theory (structural constraints), the authors provide a more nuanced explanation for why youth "choose" entrepreneurship (necessity vs. opportunity).

**3. Timely and Relevant Themes**

 The integration of AI Ethics and Digital Governance into a labour-market study is forward-thinking. It acknowledges that modern African youth enter a "platformized" labour market where algorithmic credit scoring and digital access are as important as traditional human capital.

**4. Clear Policy Implications**

 The study avoids the "blame the youth" narrative. Instead, it correctly identifies that promoting entrepreneurship without fixing systemic failures (public goods, trust, formal job creation) essentially offloads macroeconomic risk onto the most vulnerable population.

**Gaps and Weaknesses**

**1. Evidentiary Asymmetry (The Rwanda Gap)**

 While the methodological honesty is a strength, the actual comparison is weak. Sierra Leone is analysed using primary public opinion data (micro-level), while Rwanda is analysed using policy documents and secondary indicators (macro-level).

**The Problem:** You are comparing "what people say" (Sierra Leone) with "what the government reports" (Rwanda). These are different units of analysis and can lead to biased conclusions.

**2. Justification for Case Selection**

 The rationale for choosing Rwanda and Sierra Leone specifically is somewhat thin. While they are both policy-relevant, they represent vastly different governance models and post-conflict trajectories. The paper would benefit from a stronger "Most Different Systems" or "Most Similar Systems" design justification.

**3. Operationalization of TPB Constructs**

 The Theory of Planned Behaviours relies on Attitudes, Subjective Norms, and Perceived Behavioural Control.

**The Gap:** In the results section, the authors use "Economic Outlook" as a proxy for Perceived Behavioural Control and "Trust in the President" as a proxy for Institutional Condition. While plausible, these proxies are a stretch. The paper lacks a direct measure of whether youth feel capable of starting a business.

**4. The "Missing Jobs" vs. "Missing Skills" Debate**

 The paper leans heavily into the missing jobs thesis (Fox et al.). While likely correct, the paper dismisses the skills mismatch argument too quickly without looking at the specific vocational training data available in the cited Rwanda NISR reports.

**Detailed Methodological Critique**

**Temporal Disconnect:** The authors cite Rwanda NISR data from "Quarter 3 of 2025" and Sierra Leone data from "Round 9 (2021-2023)." This three-year gap is significant in a rapidly evolving digital economy.

**Confidence Intervals:** The authors provide approximate 95% Cis for Sierra Leone. Since they did not have direct access to the complex survey weights (as stated in 3.4), these intervals are based on simple binomial assumptions. They should explicitly state that these CIs may be narrower than they would be if the "Design Effect" (clustering/stratification) were fully accounted for.

**Visualizations:** Figure 6.1 (Heat-mapped dashboard) is a conceptual assessment. It lacks empirical grounding; it is an author-generated rubric rather than a data-driven output. This should be labelled as a "Proposed Framework" rather than a "Result."

**Recommendations for the Authors**

**1. Address the Round 8 Option**

 If symmetry is the goal, why didn't the authors use Afrobarometer Round 8 (where both Rwanda and Sierra Leone data are publicly available and merged)? A symmetric analysis of slightly older data (2019-2021) might be more scientifically robust than an asymmetric analysis of Round 9.

**2. Strengthen the Rwanda Context**

 To bridge the gap, the authors should look for proxy survey data for Rwanda from other sources (e.g., the World Bank’s High-Frequency Phone Surveys or the ILO’s School-to-Work Transition Surveys) to provide some level of micro-data parity.

**3. Define Entrepreneurship More Rigorously**

 The paper conflates self-employment with entrepreneurship. In Sub-Saharan Africa, a street vendor and a tech-startup founder are both self-employed, but their employment preferences and opportunity structures are diametrically opposed. Acknowledging this distinction would deepen the discussion.

**4. Clarify the "AI Ethics" Link**

 The inclusion of AI Ethics is interesting but feels tacked on in the discussion. To make this relevant, the authors should specifically link it to the Sierra Leone data on "Internet Use" (the proxy for digital access) mentioned in the design but not fully explored in the results.

 While the disclosure is a positive step for Open Science, the use of AI to surface literature and organize syntheses in a paper that already suffers from a lack of primary data symmetry (the Rwanda gap) is a "red flag."

**Final Verdict for Peer Reviewer**

**Recommendation: Minor Revision.**

 The paper is well-written and conceptually sophisticated. Its primary flaw is the lack of data symmetry, but the authors turn this into a methodological feature regarding transparency. As long as the conclusions remain cautious as promised, the paper provides a valuable contribution to the waithood and youth employment literature in Africa.

Is the work clearly and accurately presented and does it cite the current literature?

Partly

If applicable, is the statistical analysis and its interpretation appropriate?

Yes

Are all the source data underlying the results available to ensure full reproducibility?

Yes

Is the study design appropriate and is the work technically sound?

Partly

Are the conclusions drawn adequately supported by the results?

Yes

Are sufficient details of methods and analysis provided to allow replication by others?

Yes

Reviewer Expertise:

Entrepreneurship; Entrepreneurship Education; Small Business Development; Strategic Management;

I confirm that I have read this submission and believe that I have an appropriate level of expertise to confirm that it is of an acceptable scientific standard.
